# Nanolesions induced by heavy ions in human tissues: Experimental and theoretical studies

**DOI:** 10.3762/bjnano.3.64

**Published:** 2012-07-25

**Authors:** Marcus Bleicher, Lucas Burigo, Marco Durante, Maren Herrlitz, Michael Krämer, Igor Mishustin, Iris Müller, Francesco Natale, Igor Pshenichnov, Stefan Schramm, Gisela Taucher-Scholz, Cathrin Wälzlein

**Affiliations:** 1 Frankfurt Institute for Advanced Studies (FIAS), Ruth-Moufang-Str. 1, 60438 Frankfurt am Main, Germany; 2 Institut für Theoretische Physik, Johann Wolfgang Goethe-Universität, Max-von-Laue-Str. 1, 60438 Frankfurt am Main, Germany; 3 GSI Helmholtzzentrum für Schwerionenforschung GmbH, Planckstr. 1, 64291 Darmstadt, Germany; 4 Technische Universität Darmstadt, Institut für Festkörperphysik, Hochschulstr. 8, 64289 Darmstadt, Germany; 5 National Research Center "Kurchatov Institute", 1, Akademika Kurchatova pl., Moscow, 123182, Russia; 6 Institute for Nuclear Research, Russian Academy of Sciences, 7a, 60th October Anniversary prospect, Moscow 117312, Russia; 7 Center for Scientific Computing, Johann Wolfgang Goethe-Universität, Max-von-Laue-Str. 1, 60438 Frankfurt am Main, Germany

**Keywords:** DNA repair, heavy ions, microdosimetry, Monte Carlo simulations, nanolesions, radiation-induced nanostructures

## Abstract

The biological effects of energetic heavy ions are attracting increasing interest for their applications in cancer therapy and protection against space radiation. The cascade of events leading to cell death or late effects starts from stochastic energy deposition on the nanometer scale and the corresponding lesions in biological molecules, primarily DNA. We have developed experimental techniques to visualize DNA nanolesions induced by heavy ions. Nanolesions appear in cells as “streaks” which can be visualized by using different DNA repair markers. We have studied the kinetics of repair of these “streaks” also with respect to the chromatin conformation. Initial steps in the modeling of the energy deposition patterns at the micrometer and nanometer scale were made with MCHIT and TRAX models, respectively.

## Introduction

In a low-dose field of γ-rays, such as that normally experienced on Earth due to background radiation, each human cell is traversed by very few electrons, which hence produce little damage. However, for energetic heavy ions, the situation is different. A low dose, such as the one experienced in a manned mission to the International Space Station or the Moon [1], corresponds to only a few tracks, but each track can affect a whole tissue or organ, and each cell that is found in the path of the ion. The central part of the track, where most of the energy is deposited, has a radial extension of only a few nanometers, while a lower energy is deposited at a larger distance by energetic δ-rays. Thus, each heavy ion will produce a nanochannel in neighboring cells in a tissue or organ, a situation that makes the concept of low dose itself flawed. Although the concept of a “microlesion” induced by heavy ions in space was already acknowledged long ago [2], there is a lack of experimental models for testing the hypothesis that they represent a distinct, unique type of damage at the tissue level. Moreover, Monte Carlo codes should be able to simulate the damage at the micrometer and even nanometer level, basing on the stochastic energy deposition pattern. One problem associated with the formation of nanolesions is the nonuniform structure of the target, i.e., of the cell nucleus [3]. In fact, the compact heterochromatin provides a different environment compared to the transcriptionally competent euchromatin, and it had been proposed that heterochromatin was “refractory” to repair proteins [4]. We have investigated in detail the structure of nanolesions, their formation and movement in the cell nucleus, using live cell microscopy and immunohistochemistry. Stimulated by the differences in repair kinetics and movement of the tracks in eu- and heterochromatin, we have further analyzed the histone modifications (particularly acetylation) along heavy-ion nanolesions. We have also started a full-genome deep-sequencing approach to correlate the microscopy data with the cellular response. In principle, the nanolesion structure can be predicted by accurate Monte Carlo simulations of the energy deposition by the projectile and of the target structure. We used the Monte Carlo model for heavy-ion therapy (MCHIT) code [5] to simulate the energy deposition to micrometer-sized objects, e.g., cell nuclei, and compared the results to microdosimetric spectra previously measured [6]. To further describe the nanometer region, the GSI track structure Monte Carlo code TRAX [7], whose purpose is to properly describe the creation and transport of low energy electrons, has been extended to describe inhomogeneous targets.

## Results and Discussion

### 

#### Nanolesions in different regions of the chromatin

**Figure 1:**
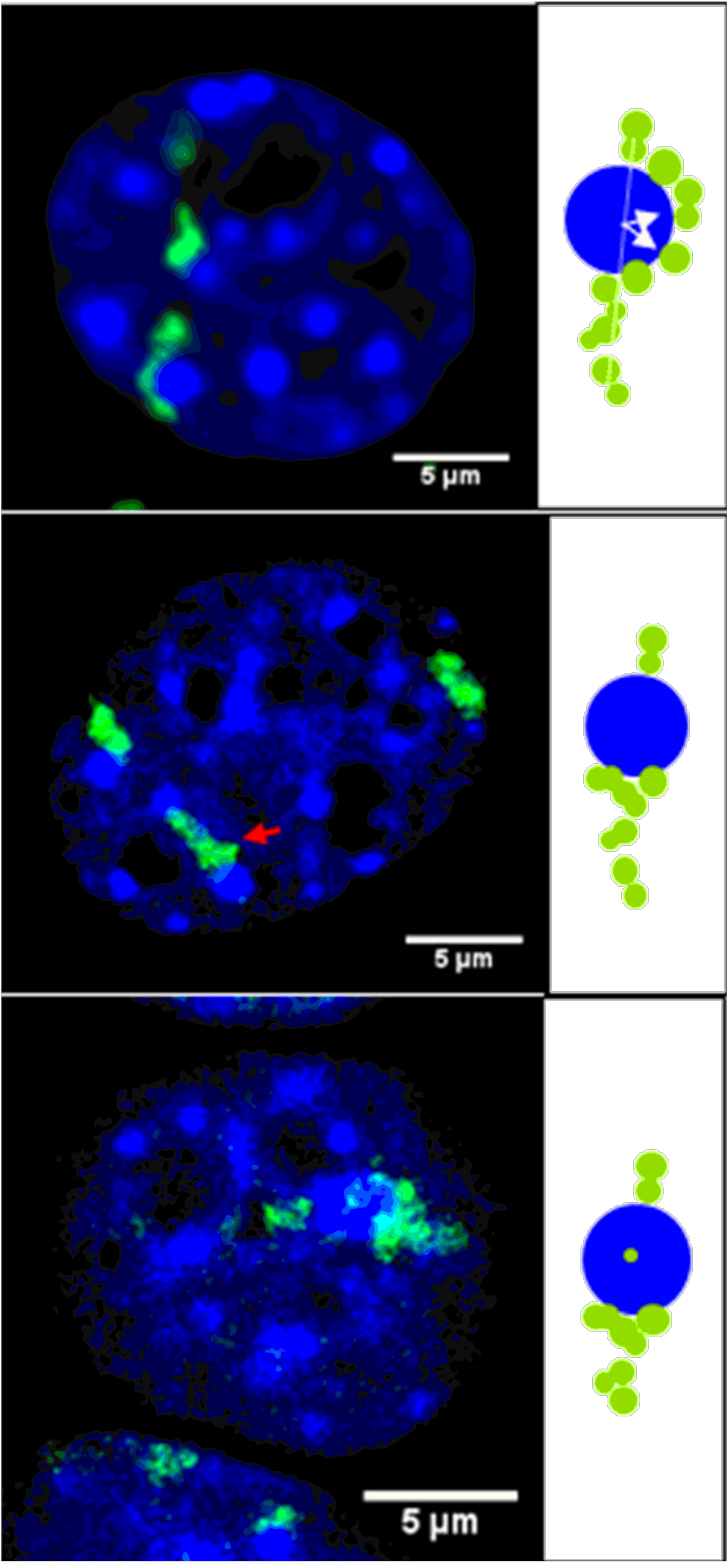
Bending of linear ion-induced γH2AX streaks indicates chromatin density-dependent damage relocation. Three types of γH2AX patterns, each shown in a mouse embryo fibroblast nucleus and as a schematic drawing, were observed at ion-hit chromo centers: bent streaks (upper panel), interrupted streaks (middle panel) and internal signals (lower panel). Modified from [8].

**Figure 2:**
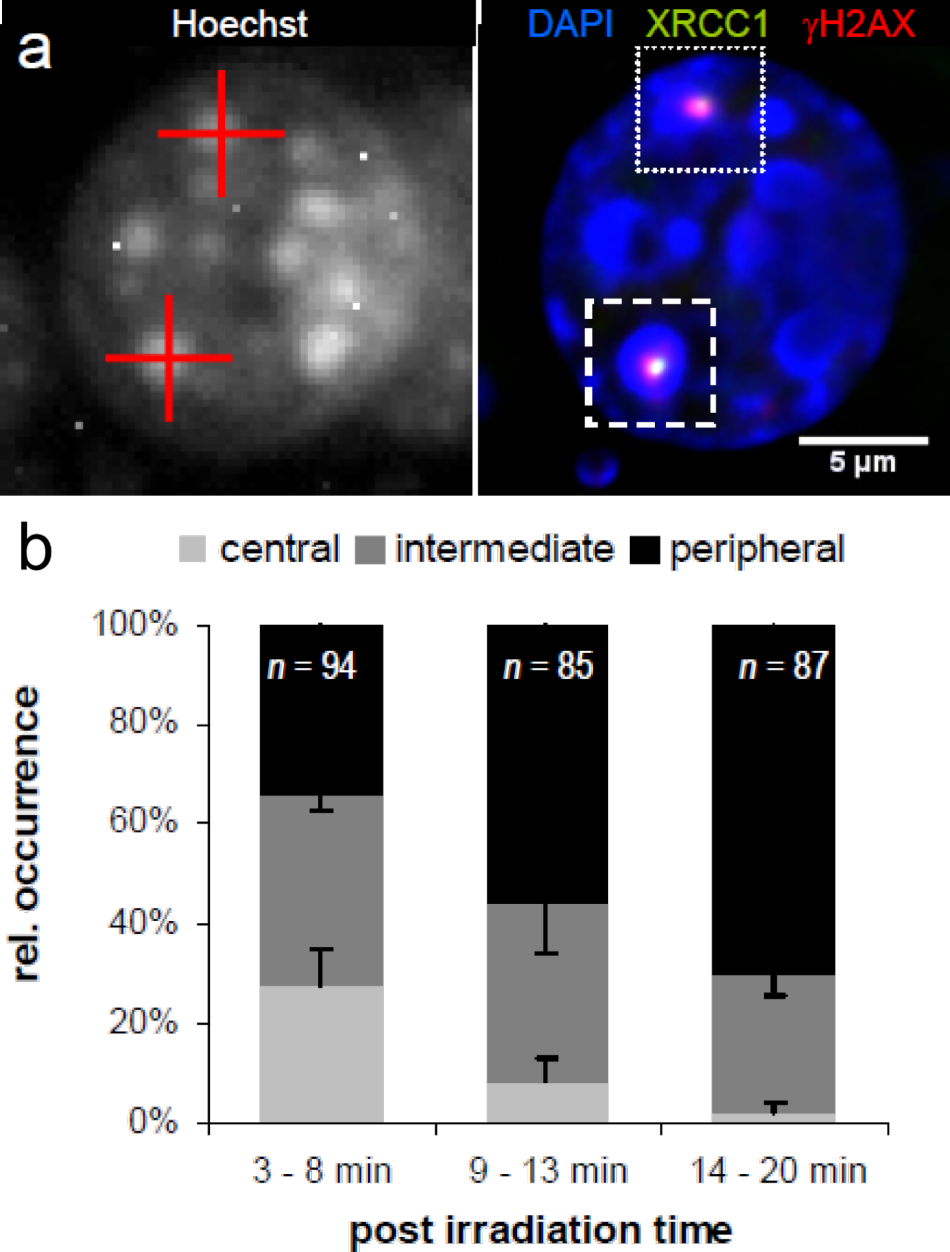
Relocation dynamics of damage sites centrally induced within heterochromatic chromo centers. (a) The mouse embryo fibroblast (MEF) nucleus was irradiated with single sulfur ions and immunostained 5 min after irradiation. H2AX is phosphorylated and the repair protein XRCC1 accumulates at heterochromatic DSBs directly after single-ion irradiation. The left-hand image shows the aimed targeting of chromo centers (red crosses) for single-ion irradiation by using Hoechst 33342 (grey scale) as a marker in the nuclei of living MEF cells. The right-hand image shows the same nucleus after fixation at 5 min after irradiation. DNA-damage-induced foci of the repair factor XRCC1 (green) and γH2AX (red) are clearly visualized at the sites of ion traversal. Both proteins colocalize within each of the targeted chromo centers (blue: DAPI DNA staining). (b) Analysis of the time-dependent localization of XRCC1 and γH2AX radiation-induced foci. Relative frequencies of each position are given for the indicated post-irradiation intervals and (n), the total number of ion-hit chromo centers from three independent experiments, is indicated. Error bars represent the SEM. Figure adapted from [8].

Physics obviously predicts that streaks produced by heavy ions in the DNA should be linear. However, using a double-strand break (DSB)-specific marker (phosphorylated histone γH2AX), we found “bending” of the streaks when cells were fixed for 30 min or more after irradiation [8] (Figure 1). Reconstruction of the track dynamics by using live-cell imaging (Supporting Information File 1) and the heavy-ion microbeam (Figure 2) showed that DNA-DSBs are indeed formed within heterochromatin, but they are relocated to euchromatin and the repair kinetics are slower than for euchromatic lesions [8]. These results in mammalian cells have also been observed at the Lawrence Berkeley Laboratory in *Drosophila* [9], thus suggesting that the lesion relocation from hetero- to euchromatin is a universal phenomenon.

#### Conformational changes in chromatin

**Figure 3:**
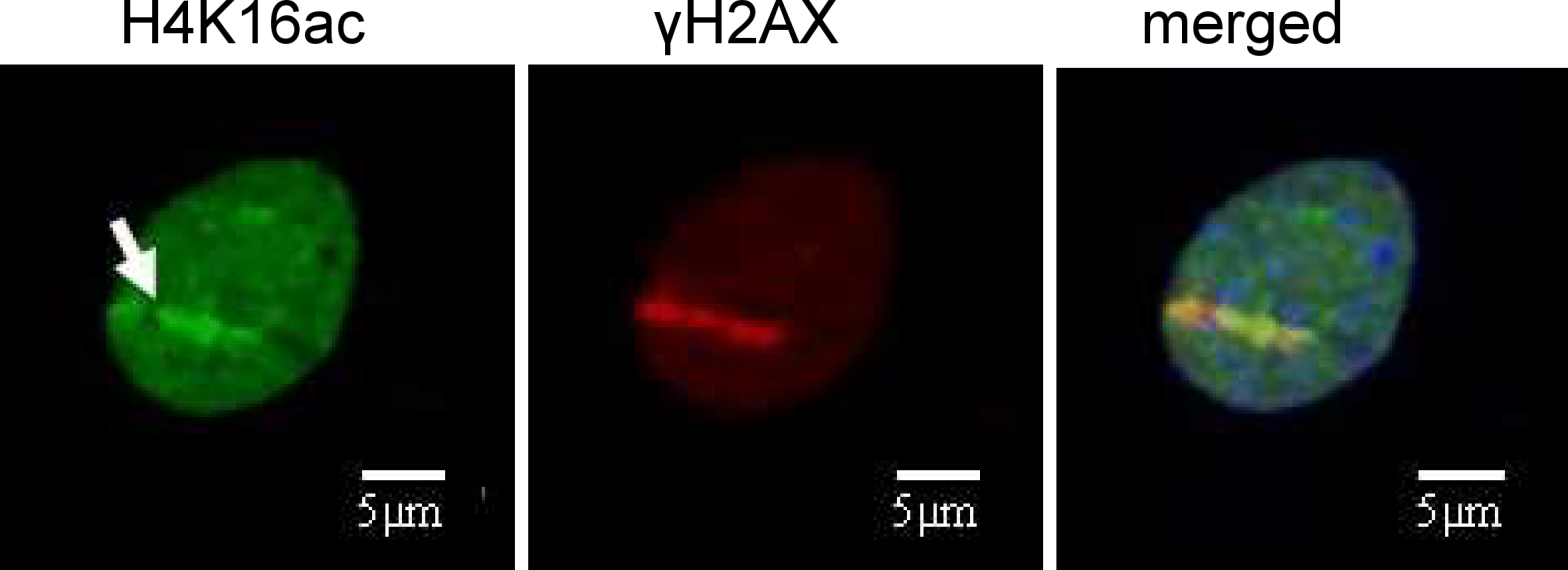
Accumulation of H4K16ac in mouse embryonic fibroblasts. Cells were irradiated with Au ions (energy: 8 MeV/n, linear energy transfer (LET): 13000 keV/μm; fluence: 3·10^6^ ions/cm^2^) at a low angle and fixed after 1 h. H4K16ac (green) is increased at damage sites. DNA damage is shown by γH2AX staining (red). DNA is counterstained with ToPro3 (blue). From [12] – Copyright: GSI Helmholtzzentrum für Schwerionenforschung GmbH.

The results described above point to a large-scale chromatin decondensation at sites of nanometric DNA lesions. This observation shifted our attention from the analysis of “DNA nanolesions” to a more general concept of “chromatin nanolesions”. Histone modifications, especially histone acetylation at defined lysine residues, play a major role in changing the density of chromatin. To explain the local decompaction of heterochromatic regions that takes place at sites of DNA damage [8], we investigated the acetylation of different histone residues that may be involved in this process. We investigated the histone residues H4K16 as well as H3K56. It is known that these residues play a role in the DNA damage response after irradiation by X-rays and UV-lasers [10–11]. In a limited fraction of cells, we measured H4K16ac streaks after exposure to heavy ions (white arrow in Figure 3) that are clearly distinguishable from the H4K16ac signal in the whole nucleus [12].

An accumulation of H3K56ac was not observed. These findings suggest that H4K16ac may also play a role in the damage response after irradiation with heavy ions. Since it is known that acetylation of H4K16 changes chromatin to a more open conformation it has to be elucidated whether H4K16 acetylation is involved in decompaction of DNA at damage sites. However, not all of the cells presented visible H4K16ac streaks, and the effect was observed only with very heavy Au ions. At the fluence used in our experiments (3·10^6^ Au-ions/cm^2^) we measured an average of three streaks/cell by using DNA repair markers, and therefore less than 5% of the cells should have no streaks according to Poisson statistics. Moreover, experiments with lighter ions did not produce clear signals. Further experiments are underway to clarify these issues.

#### Genome-wide screening of the chromatin nanolesions

**Figure 4:**
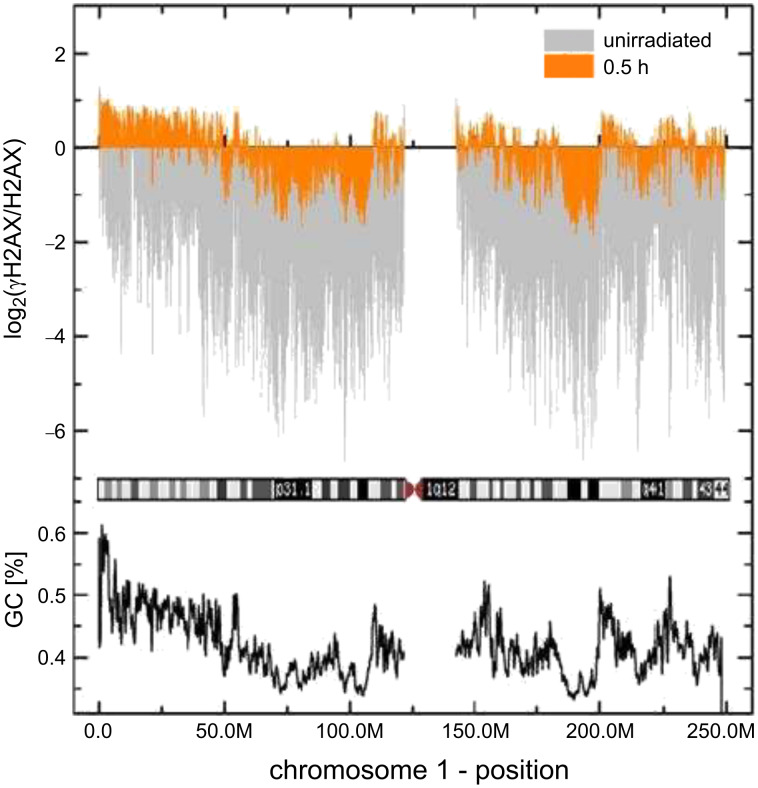
The phosphorylated H2AX distribution after radiation is correlated with the GC base content (a genomic feature associated with high gene content) of the transcriptionally competent and relaxed chromatin (euchromatin). The chromosome 1 profile is shown in the cartoon, with dark bands corresponding to heterochromatin and light bands to euchromatin (from chromosome G-banding). Preliminary ChIP-Seq data show that the γH2AX signature (orange) is enriched in high-GC-content DNA sequences (black line, below) and dark chromosomal bands (e.g., p31.1, q41), corresponding to a very low GC content are underrepresented. From [14] – Copyright: GSI Helmholtzzentrum für Schwerionenforschung GmbH.

Alternatively to observation by microscope, the distribution of DNA nanolesions can be investigated with the novel ChIP-Seq technology [13], which allows the mapping of DNA-protein interactions sequence-wise and genome-wide. We used ChIPSeq to provide a genome-scale sequence-based map of the γH2AX signature induced by ionizing radiation. Compaction state of chromatin domains was characterized by multiparametric analysis (e.g., GC content), and the distribution of radiation-induced γH2AX along such chromatin domains was investigated. This complex study is still underway, but preliminary results (Figure 4) suggest that γH2AX is positively correlated to the GC content. Such a feature would indicate that a less compact state (high GC content) could be a more favorable environment for γH2AX spreading than highly compact heterochromatin [14].

#### MCHIT simulations of microdosimetry distributions

The Monte Carlo method is a convenient technique to account for the interactions of beam nuclei and all secondary particles with tissues. The MCHIT [5] based on the Geant4 toolkit was created in the Frankfurt Institute for Advanced Studies (FIAS) to study the propagation of therapeutic beams in extended media. MCHIT calculates the spatial distribution of energy deposited in a tissue-like phantom, taking into account the fragmentation of beam nuclei.

A practical way to investigate energy deposition to objects equivalent to living cells consists of measurements with detectors called tissue-equivalent proportional counters (TEPC). Typically a TEPC is designed as a low-pressure gas chamber a few millimeters in size. The energy ε delivered to the small sensitive volume in a single event fluctuates due to the stochastic nature of particle propagation in media. Microdosimetry measurements provide the probability distributions for lineal energy defined as *y* = ε/<*l*>*,* where <*l*> is the mean chord length of the sensitive volume of the detector. The distributions of lineal energy (microdosimetric spectra) are directly related to the biological effects of radiation.

**Figure 5:**
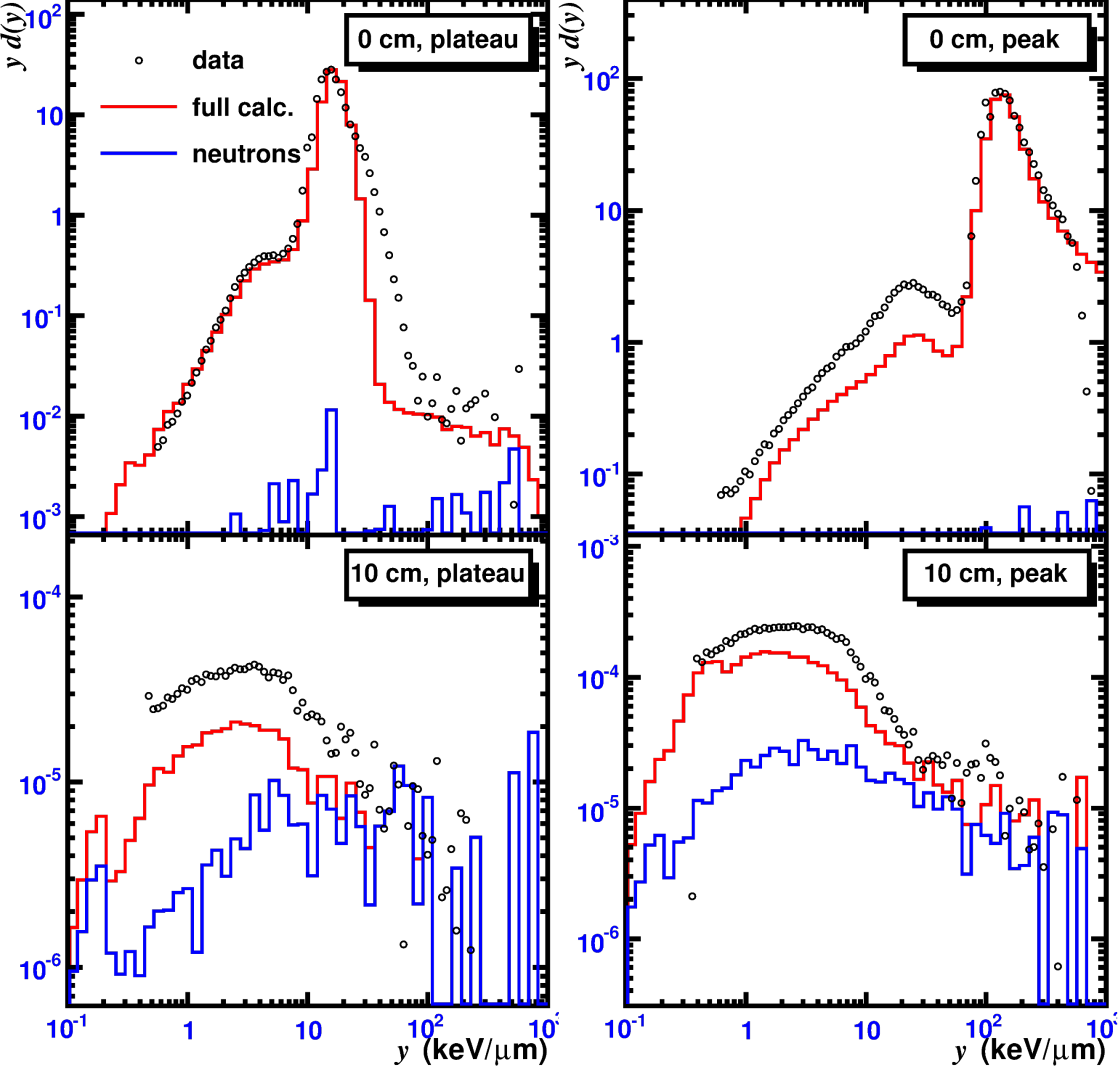
Microdosimetric spectra in a water phantom irradiated by 300 A MeV ^12^C nuclei. Upper (red) histograms are the total spectra calculated with MCHIT, the lower (blue) histograms show the neutron contributions. Data points from [6].

The MCHIT model was used to simulate microdosimetry measurements at GSI [6]. In this experiment the microdosimetry *yd*(*y*) spectra (see [6] for their definition) were collected on the beam axis, as well as off-axis, inside a water phantom, irradiated by a narrow 300 A MeV ^12^C beam. Simulation results for four TEPC positions inside the phantom are shown in Figure 5. Two of the four measurements (marked as “0 cm”) were performed on the beam axis and the other two at 10 cm radius at the beam entrance to the water phantom (“plateau”) and at the depth of the Bragg peak (“peak”).

**Figure 6:**
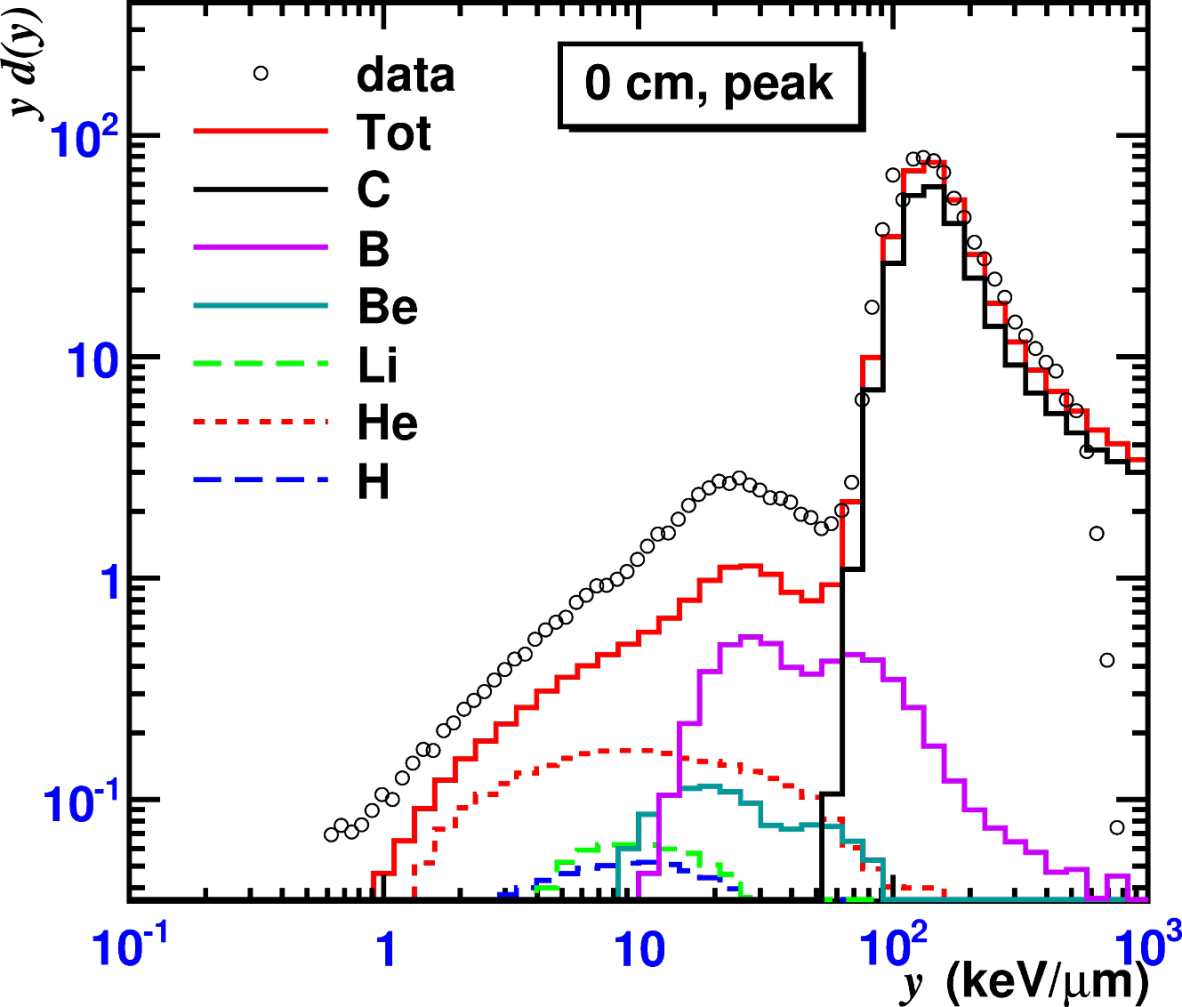
Contribution of various fragments to the microdosimetry spectra measured on the beam axis in the Bragg peak region in a water phantom irradiated by 300 A MeV ^12^C nuclei. Histograms represent MCHIT calculations. Data points from [6].

It is known that secondary particles of various charges and velocities can eventually contribute with similar lineal energy values. Therefore, the considered *yd*(*y*)*-*distribution is built as a sum of contributions from various secondary particles representing a multicomponent radiation field around the primary beam. The contributions of various fragments to the spectra are shown separately in Figure 6 for a TEPC located at the Bragg peak on the beam axis. The peak in the distribution at *y* ≈ 131 keV/μm is due to the primary carbon nuclei while the second broad peak at *y* ≈ 25 keV/μm is caused by projectile fragments produced in fragmentation reactions. The MCHIT model reproduces the general shape of *yd*(*y*) distributions at all four TEPC positions in the phantom (Figure 5). However, it underestimates the spectra for TEPCs located far from the beam axis. This problem is apparently related to an underestimation of yields of light fragments produced by primary nuclei in the phantom. The contributions to *yd*(*y*) distributions from secondary neutrons are also shown in Figure 5 for the considered TEPC positions. The neutron contribution increases with the distance from the beam axis. At the TEPC positions far from the beam (at 10 cm radius) the total contribution from neutrons amounts to ≈50% at the plateau and to ≈25% at the Bragg peak depth. More details on microdosimetry simulations with the MCHIT model, in particular on specific physics models used in calculations, can be found elsewhere [15].

#### Extensions of the TRAX code

The elevated radiobiological effectiveness of heavy ions can be attributed to the largely inhomogeneous damage deposition on the micro- or even nanometer level when compared to sparsely ionizing reference radiation with the same macroscopic dose deposition. Whereas photons or electrons show an almost uniform distribution of interaction events, even on this small-scale level, the dose deposition caused by ions is centered on the track core and falls off as 1/*r*^2^. Thus, towards the ion-track core, local doses deposited by ions can reach values up to kilo- or even megagrays for the heaviest ions. On the other hand, biological endpoints important for radiotherapy, such as tumor cell killing and healthy tissue damage, follow the well-known and well-proven linear-quadratic dose dependence. This means that high doses, as they occur in the ion track core, contribute disproportionally to the radiobiological effect. Thus this part of the radial dose distribution will contribute most to the radiation action. Unfortunately the ion track core is also the least known region in this scenario. Available models usually cut off and renormalize the radial dose at distances of the order of ten nanometers to avoid the mathematical divergence at *r* = 0, which is justified by reasonable results, but somewhat unsatisfying from the physical point of view. Experimental data are almost nonexistent in this region, even in gases, let alone in condensed phase.

**Figure 7:**
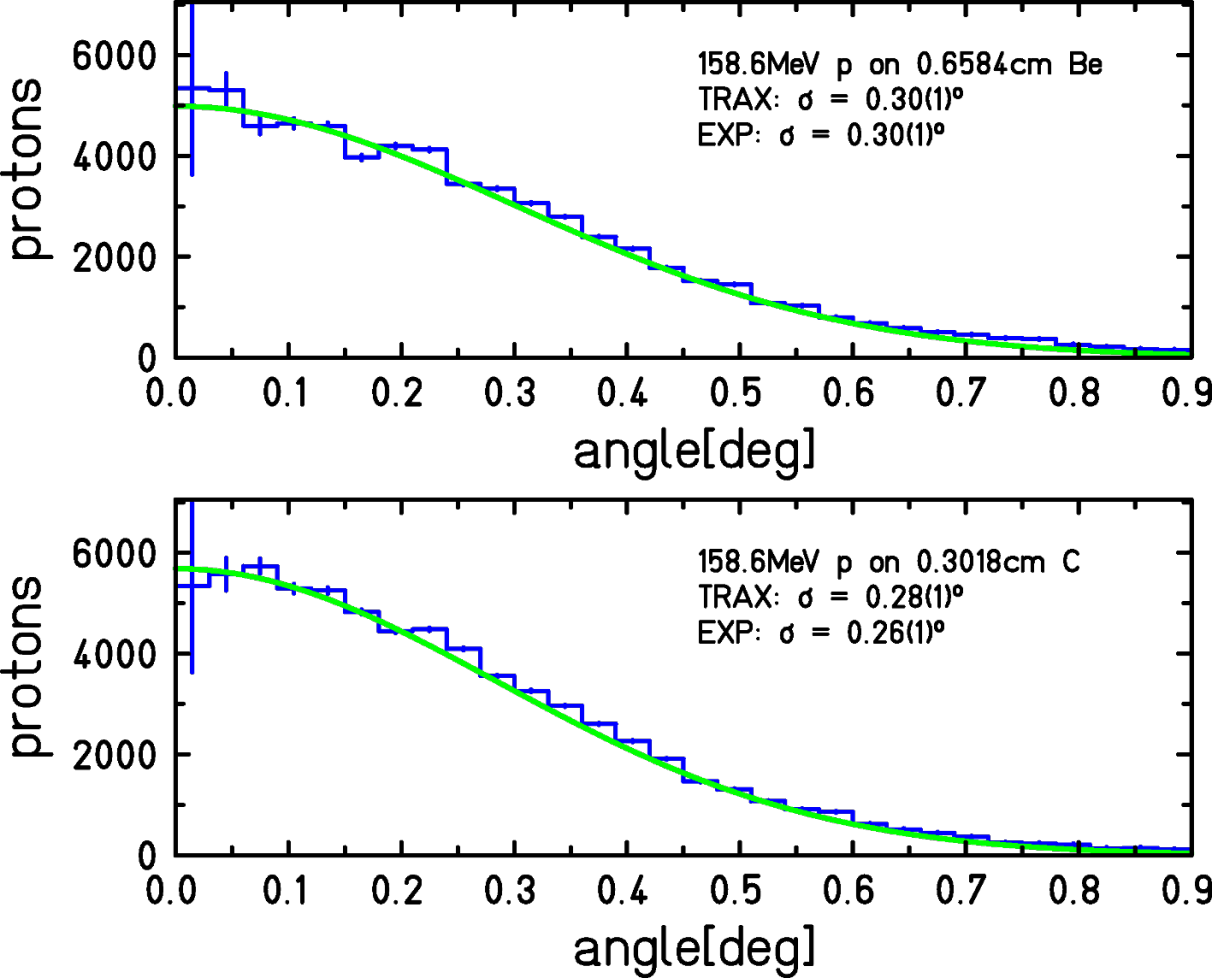
Simulated angular distributions of 158.6 MeV protons incident on 0.66 cm beryllium (upper picture) and 0.30 cm carbon (lower picture) with TRAX. The binning of the histogram in the TRAX simulation is 0.03 degrees. In the case of the beryllium target, fitting a Gaussian distribution to the simulated data resulted in a Gaussian width of (0.30 ± 0.01)° which is exactly the same result as experimentally determined in [17]. Highland's formula [18] led to a width of 0.27°. In the case of carbon, the fit to the TRAX results resulted in a Gaussian width of (0.28 ± 0.01)°, while Gottschalk et al. [17] determined this width to be (0.26 ± 0.01)°. Highland's formula [18] resulted in 0.24° for the carbon target.

To improve on this situation, at least from the computational side, we apply our simulation code TRAX [7], constantly developed at GSI over several years. It uses the single interaction Monte Carlo method, rather than a condensed random walk, to describe radiation action at the lowest possible level. When inspecting the nanoscale, however, not only the usual ionization and excitation events, but also elastic scattering of the primary ion, which is often neglected, may play a role. Therefore we have included this interaction in the simulation to evaluate its influence on the nanoscale damage distribution. Screened Rutherford cross sections according to Berger [16] were used to account for the elastic scattering of ions. The correct implementation of this additional interaction in the code was benchmarked against experimental results. Gottschalk et al. [17] have measured the angular distribution of 158.6 MeV protons incident on several different target materials and thicknesses. The TRAX simulations including elastic ion scattering showed good agreement with these experimental results, as can be seen in Figure 7.

**Figure 8:**
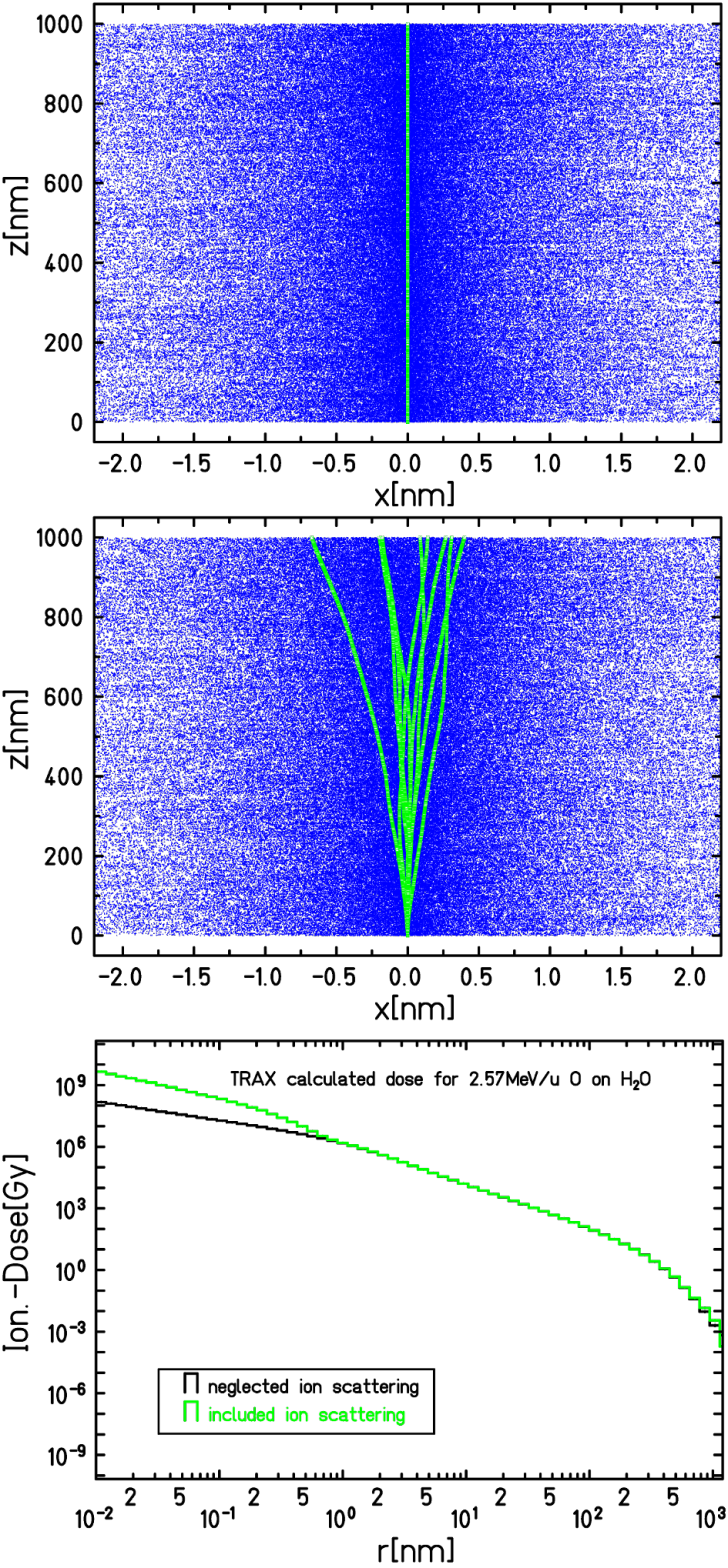
In the upper picture, the tracks of 10 individual oxygen ions with a primary energy of 2.57 MeV/u, incident on water, are shown while the elastic scattering of ions is neglected. They all travel straight through the medium. The blue spots indicate the interaction positions of the secondary electrons. In the central picture the same plot is shown including the elastic scattering of ions. The angular deflection over a travelling length of 1 μm can be seen. On the nanometer scale, the shift of ionization and excitation events of the primary ions is noticeable. The resulting radial dose with and without elastic ion scattering is shown in the lower picture. It can be seen that the radial dose differs in the area that is equal to the radius of the elastic scattering of ions. The deflection of the primary ions leads to a natural "diffusion" of the radial dose.

Additionally the simulations were compared to Highland's formula [18], which is a parameterized approximation of the Molière theory. The implementation of elastic ion scattering is an important step towards a complete description of the relevant physical effects that contribute to the energy deposition on the nanoscale. However, further extensions of the code may be necessary to account for all important physical effects. Figure 8 shows that the elastic scattering of the primary ions has an effect on the nanometer scale. Energy deposition events, such as excitation and ionization, which are caused by the primary ions, no longer occur only at *r* = 0. The positions are shifted on the nanometer scale. This reduces the calculated radial dose at *r* = 0 (not shown in the figure) and increases the calculated radial dose at radii within the scattering radius of the primary ions.

## Conclusion

We have developed experimental techniques to visualize nanolesions in human tissues and to analyze these lesions genome-wide. In our approach, nanolesions are induced by very heavy ions and studied by the recruitment of repair proteins and the epigenetic changes in the chromatin surrounding the damaged DNA molecule. We concluded that the structure of the nanolesions depends strongly on the target structure, where the target is not only DNA, but the protein-nucleic acid complex (chromatin). Monte Carlo codes MCHIT and TRAX can elegantly reproduce the measured [6,17] energy deposition patterns following the passage of energetic heavy ions. However, further efforts are required to improve the MCHIT model accuracy in calculating spectra far from the beam axis and to extend TRAX to complex inhomogeneous targets. Novel target simulations will be necessary to simulate the observed formation and dynamics of nanolesions in tissues. Further extensions of the MCHIT and TRAX code will be necessary to obtain a satisfactory description of energy deposition and track behavior at the nanometer scale in realistic targets.

## Experimental

**Figure 9:**
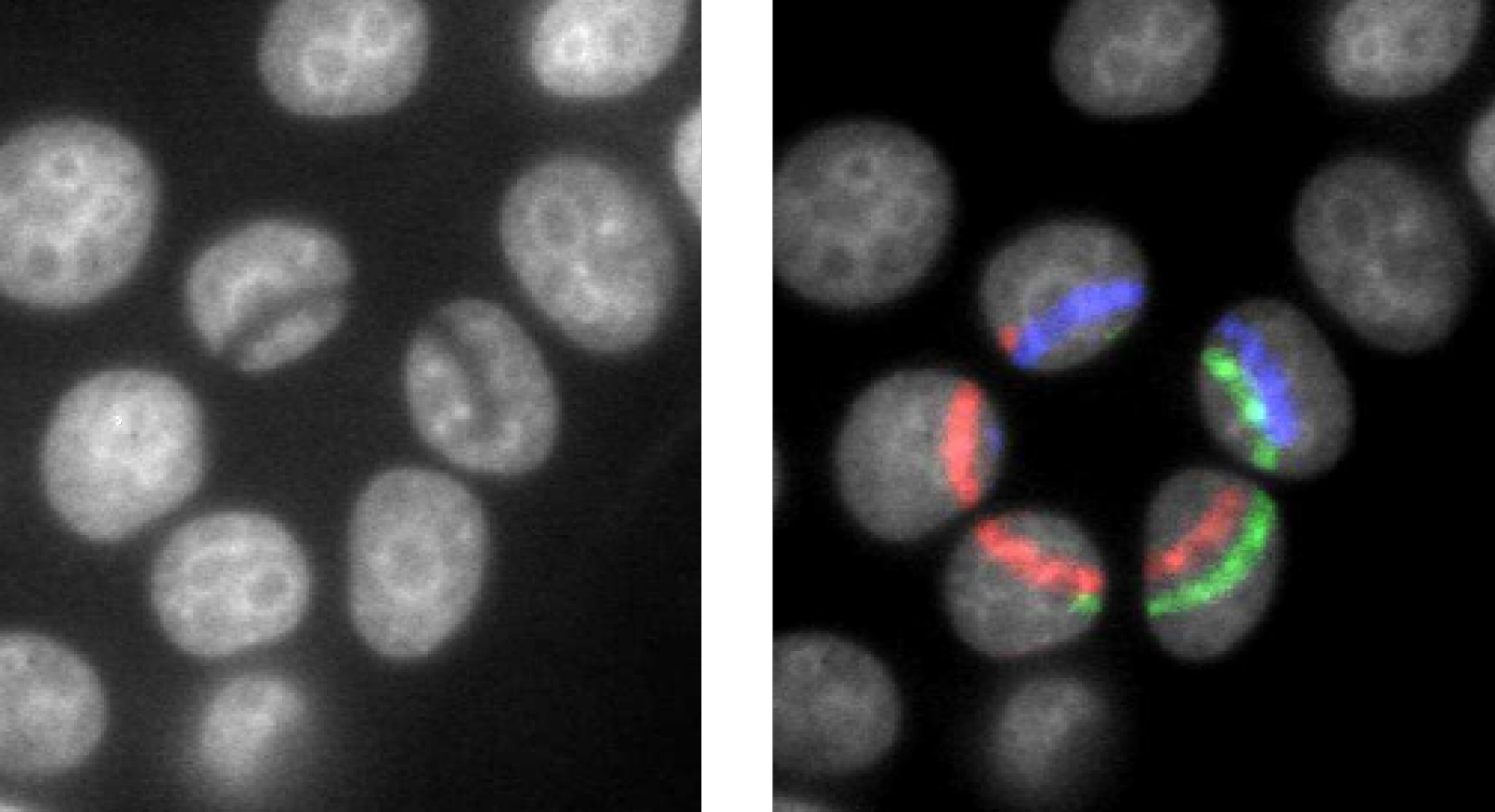
Living HeLa cells expressing histone H2B tagged to GFP were photobleached. Bleaching within a region of three sectors of a circle depletes fluorescence from the bleached region. Colors of the three regions were adjusted with ImageJ and the different channels were merged. From [19] – Copyright: GSI Helmholtzzentrum für Schwerionenforschung GmbH.

Detailed experimental methods for immunohistochemistry and live-cell imaging in our laboratories are described elsewhere [8,12]. We have recently installed a 405 nm laser for photoactivation studies [19]. The experimental setup is based on a Leica IRE2 inverted microscope equipped with LED light sources and a climate chamber for controlling of the temperature, humidity and CO_2_ concentration, for long-term live-cell observations. Image acquisition is done by a Hamamatsu C7190 EB-CCD camera. Photobleaching of GFP-tagged H2B in living HeLa cells by the 405 laser is demonstrated in Figure 9. By turning and panning of the laser circle, the logo of the Beilstein-Institut was visualized by pseudocoloring of the bleached regions. Details of the microdosimetry measurements are given in [6]. We simulated with MCHIT the TEPC model LET-1/2, Far West Technology at a gas pressure of 120 mbar, equivalent to 2.7 μm tissue.

## Supporting Information

File 1 The animation in Supporting Information File 1 shows a real time observation of the recruitment of GFP-XRCC1 to two charged particle tracks traversing the nucleus of a living MEF cell during high energy (1 GeV/n) uranium irradiation. From these 3-D image stacks, movies were generated by making maximum projections of the fluorescence intensity using Image J (http://rsb.info.nih.gov/ij/). Red color indicates Cherry-tagged HP1α (marking chromocenters), green color GFP-XRCC1. Total imaging time: 9.5 min. Shot noise (due to neutron scattering) indicates the irradiation time points. Please note the fast GFP-XRCC1 recruitment along tracks, disappearance of euchromatic foci (green) and the prolonged retention of heterochromatic GFP-XRCC1 (yellow, overlapping HP1α) in the left radiation track.

File 2 Supporting Information File 2 is a high resolution animation showing real time GFP-XRCC1 recruitment to the high energy uranium ion track traversing a single MEF chromocenter (red, marked by Cherry-HP1α). Note the billowing motion of the damaged domain (XRCC1, green; appears yellow due to HP1α overlap in heterochromatin) and a drift toward the chromocenter periphery.
